# Clinical Significance and Expression of PAF and TNF-alpha in Seminal Plasma of Leukocytospermic Patients

**DOI:** 10.1155/2012/639735

**Published:** 2012-11-06

**Authors:** Chaodong Liu, Hongjian Liu, Xianzhong Wang, Sun Xinbo

**Affiliations:** Department of Urology, First Affiliated Hospital of Chongqing Medical University, Chongqing 400016, China

## Abstract

*Objective*. Discuss the changes and roles of PAF in the reproductive tract infection by observing the expression of platelet activating factor (PAF) and tumor necrosis factor **α** (TNF-**α**) in seminal plasma of patients with leukocytospermia. *Methods*. The seminal plasma was obtained from 22 cases of leukocytospermia and 15 cases of normal males; the peroxidase dyeing method was adopted for seminal plasma white blood count; the ELISA was adopted to test PAF and TNF-**α** concentration in seminal plasma. *Result*. PAF concentration (2.14 ± 0.43 ng/mL) of leukocytospermia group was significantly lower than the normal group (6.21 ± 1.38 ng/mL, *P* < 0.01) while TNF-**α** (5.51 ± 1.46 ng/mL) was significantly higher than that of normal group (3.48 ± 1.08 ng/mL). There was negative correlation between PAF and TNF-**α** , (*r* = −0.68, *P* < 0.01); the same situation existed in PAF and WBC (*r* = −0.62, *P* < 0.01); but TNF-**α** was positively correlated to WBC (*r* = 0.77, *P* < 0.01). *Conclusion*. (1) Low expression of PAF and high expression of TNF-**α** in leukocytospermia affect the sperm motility, which is one of the reasons that leads to infertility. (2) Lower expression of PAF has its particularity during the reproductive tract infection.

## 1. Introduction

Leukocytospermia is a kind of common disease of male reproductive tract infection, which is usually caused by bacterial infection. An increase of white blood cells in seminal plasma can lead to the decrease of sperm quality and the increasing number of inflammatory cytokines such as interleukin IL-6 is the main factor involved in the process [1]. According to current studies, PAF is a kind of cytokine which has many kinds of biological activities, such as inflammation factors and sperm cell activation factors. How does the PAF change and what role does it play the role during the reproductive tract infection? The purpose of our study was to observe the expression of PAF and TNF-*α* in seminal plasma of patients with leukocytospermia.

## 2. Material and Method 

### 2.1. Research Population

Male patients age from 23 to 40 years old, who came to the Urology Surgical Department of our hospital from April to September 2011, were enrolled. According to WHO directives, 22 cases with leukocytospermia and 15 normal males were chosen. All the patients did not have any urinary, reproductive, blood, or endocrine diseases. The patients who have ever suffered from mumps, cryptorchidism and sexual dysfunction and ever accepted radiotherapy or chemotherapy were excluded. All the indexes were in the normal range according to seminal plasma regular analysis. The study was admitted by the local ethical committee, and written informed consent was obtained from all participants.

### 2.2. Specimen Collection and Processing

Volunteers should accept abstinence for seven days firstly. Then, hands and penis should be washed with soap before the specimen collection. The semen should be obtained in one time by using masturbation method and be placed into disposable sterile dry plastic container with cover. The container was put into a water-bath box in 37°C constant temperature. After the full liquefaction of semen plasma, the computer-assisted semen analysis (CASA) carried out a routine analysis. The rest of the seminal plasma was collected and accepted centrifugal test for 10 minutes at 4000 r/min, the upper layer of seminal plasma was placed into Eppendorf tube and stored at −20°C for reservation. Density count of seminal plasma white blood cell in seminal plasma refer to peroxidase dyeing method, recommended by the WHO.

### 2.3. Main Reagent and Equipment

Human platelet activation factor (PAF) enzyme league immune kit and human tumor necrosis factor (TNF-*α*) enzyme league immune kit were bought from Jiahui Biotechnology Company (RD import repacking), Beijing Weili WJY9000 sperm analyzer, low temperature centrifuge (SIGMA, USA), 37°C constant temperature water-bath box, full-wavelength enzyme standard instrument (Finland Leibo Company). 

### 2.4. ELISA Test

The operator should operate strictly in accordance with the manual of ELISA kit for inspecting TNF-*α* and PAF. The OD value should be read on Enzyme standard instrument within 15 minutes at 450 nm wavelength.

### 2.5. Statistical Methods

The experimental data were expressed by means ± SD. SPSS (version 17.0) statistical software was used for processing the data. Normal characteristic test and homogeneity test of variance would be carried out before intragroup comparison of PAF and TNF-*α* expression, and *t*-Student test was adopted. There are two kinds of correlation analyses here, straight line related analysis and Spearman rank correlation analysis, which one should be adopted, it depends on whether the expression of PAF or TNF-*α* on normal distribution or not in the seminal plasma. *P value* less than 0.05 was considered to statistically significant.

## 3. Results

### 3.1. Results of Main Parameters in Seminal Plasma Conventional Analysis

Significant difference was found in the aspects of sperm survival rate and vigor between leukocytospermia group and normal group, *P* < 0.01, while the difference in sperm density and liquefaction time was not obvious, *P* > 0.01 (Table 1).

### 3.2. PAF and TNF-*α* Concentration in Seminal Plasma

PAF concentration in leukocytospermia group was (2.14 ± 0.43 ng/mL), which was significantly lower than the normal group (6.21 ± 1.38 ng/mL); the difference was statistically significant (*P* < 0.01). WBC count in leukocytospermia group was (1.33 ± 0.18) × 10^6^/mL, and TNF-*α* concentration was (5.51 ± 1.46 ng/mL), which was significantly higher than that of normal group WBC (0.43 ± 0.19) × 10^6^/mL and TNF-*α* (3.48 ± 1.08 ng/mL), and the difference was statistically significant (*P* < 0.01) (Table 2). 

### 3.3. Relation between PAF, TNF-*α*, and Seminal Plasma Parameters in Leukocytospermia Group

In this group, PAF has no significant correlation with sperm density and liquefaction time. And what's more, it has positive correlation with sperm survival rate (*r* = 0.452, *P* < 0.01) and sperm vigor (*r* = 0.642, *P* < 0.01). 

TNF-*α* has no significant correlation with sperm density and liquefaction time. (*P* > 0.01). While it has negative correlation with sperm survival rate (*r* = −0.415, *P* < 0.01) and sperm vigor (*r* = −0.725, *P* < 0.01) (Table 3).

### 3.4. Relativity Analysis of WBC Count, PAF, and TNF-*α* Expression Level of Leukocytospermia Group

**Figure 1:**
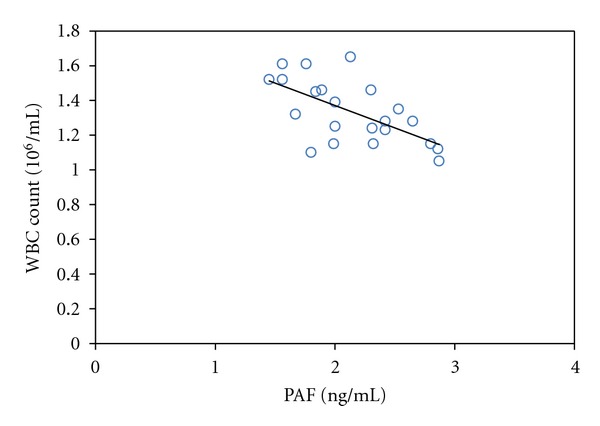
Relativity analysis of PAF and WBC count in leukocytospermia group.

**Figure 2:**
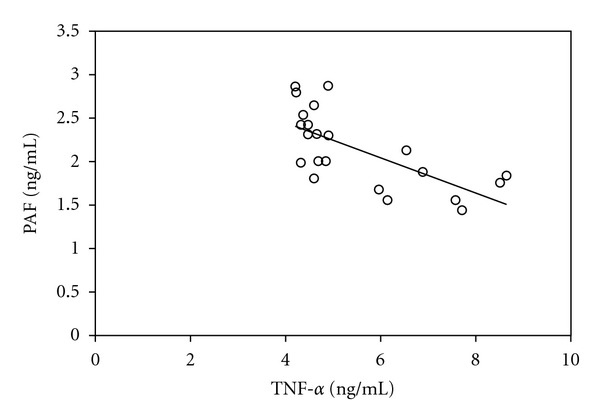
Relativity analysis of TNF-*α* and PAF in leukocytospermia group.

Linear correlation analysis shows that WBC count has negative correlation to PAF concentration in leukocytospermia group (*r* = −0.62,  *P* < 0.01) (Figure 1), however, it has positive correlation with TNF-*α* concentration (*r* = 0.77, *P* < 0.01); PAF concentration had significantly negative correlation to TNF-*α* (*r* = −0.68, *P* < 0.01) (Figure 2).

## 4. Discussion 

The reproductive tract infection is one of the common causes of clinical infertility. Leukocytospermia refers to the increase of white blood cell (WBC) density in seminal plasma which is more than 1 × 10^9^/L, and it is mainly caused by the reproductive tract infection. The white blood cells in seminal plasma can produce many inflammatory cell factors. With the affection of these cell factors, the sperm quality, density and its vigor get lower; sperm capacitation and acrosomal reaction would be affected as well, which lead to male infertility.

PAF is a kind of cell factors found in recent years, and it is involved in various physiological and pathological processes. PAF has close relationship with procreation, fetal development, and delivery [2]. Sperm movement ability is the precondition of fertilization, wherein PAF with certain concentration is considered to be one of the conditions to obtain certain movement ability. When the exogenous PAF concentration is between 1 × 10^−10^ and 1 × 10^−13^ mol/L, the sperm's motility is prominently increased after 120 min culture time [3]. In animal experiments, the sperm motility can be immediately improved after exogenous PAF treatment on freeze-thaw boar sperm [4]; PAF concentration in seminal plasma of squirrel monkeys in the breeding season is significantly higher than that during the nonbreeding season [5], thereby, it is believed that PAF plays an important role in the reproductive process. PAF also participates in sperm capacitation and acrosomal reaction (AR); sperm capacitation can be affected through culturing with sperm together, thereby increasing the acrosomal reaction rate [6]. AR% is different when PAF concentration changes, relatively, high density (1 × 10^−9^ mol/L) can induce AR better [3]. In addition, PAF can obviously increase the pregnancy rate of accepting artificial insemination treatment due to unexplained infertility [7]. The seminal plasma of dog stored in low temperature has prominently increased sperm movement performance through treatment with different concentrations of exogenous PAF. Research shows that the sperm movement performs best when the concentration is 1 × 10^−3^ M with 120 min of cultivating time, at the same time, ATP concentration improves obviously as well. We also found that the mitochondrial function of the sperm through PAF processing remains unchanged, and the integrity of sperm has not been damaged either [8]. At the same time, it promotes the platelets and neutrophils to gather together under the pathologic state participate in super oxide formation, and it also promotes the protein phosphorylation process. It can also induce submucosal vasoconstriction, and induce neutrophils and other inflammatory cells to produce a large number of active oxygen and free radicals. It is confirmed that PAF involves in gastric ulcer, alcoholic stomach injury, necrotizing enterocolitis, ischemic stomach, and small intestinal mucosal injury. In addition, it also plays an important role in acute pancreatitis and acute lung injury development [9, 10]. As a result, the role PAF which plays in reproductive tract infection is worth discussing.

Alpha subtype of tumor necrosis factor (TNF-*α*) is a kind of important cell factor involved in the inflammation reaction progress. It is mainly produced by macrophages, and widely involved in inflammation response, immune response, endotoxin sex shock, and other pathologic process. At the same time, TNF-*α* is a kind of cell factor, which has close relations with sterility occurrence and development. A certain amount of TNF-*α* can significantly inhibit the activity of sperm acrosomal enzyme and acrosomal reaction [11]. TNF-*α* has certain influence on sperm mitochondrial function, it can interfere sperm energy metabolism and lower sperm movement ability. It may also promote the sperm apoptosis through the mitochondria apoptosis way, which causes infertility [12]. TNF-*α* also can reduce the amount of nitrate in normal sperm and affects NO synthesis, which is necessary for sperm movement [13]. Studies have reported that PAF and TNF-*α* can mutually cooperate with each other to promote the progress of inflammatory response [14], such as acute lung injury [15], monilia infection [16], and necrotizing enterocolitis [17]. How is the PAF expression in the condition of reproductive tract infection? And how does the interaction happens between PAF and TNF-*α*? The mechanism is still unclear.

ELISA method was adopted to observe the expression of PAF and TNF-*α* in seminal plasma of patients with leukocytospermia. The results show that PAF level is lower, while TNF-*α* is higher compared with that in the normal seminal plasma. After analyzing the expressions of PAF and TNF-*α*, the relation between them, and the white blood count, we found that the expression of PAF and TNF-*α* have negative correlation with each other (*r* = −0.68, *P* < 0.01Figure 1); PAF is negatively correlated with white blood cell count (*r* = −0.62,  *P* < 0.01Figure 2); TNF-*α* has positive correlation to white blood cell count (*r* = 0.77, *P* < 0.01). The existing research results showed that PAF is favorable for sperm function activities, while TNF-*α* could inhibit sperm function activities. So we believe that, under condition of reproductive tract infection, the organism increase the composition of cell factors which can inhibit sperm activity on one hand, such as TNF-*α*, and decrease the composition of cell factors which can promote sperm on the other hand, such as PAF, thereby affect sperm vigor. This is one of the reasons why leukocytospermia cause infertility. PAF and TNF-*α* are considered as inflammation media and would increase in inflammatory diseases. However PAF level is lowered under the state of reproductive tract infection, this phenomenon is of great significance and worth being studied.

What is the fundamental cause of the PAF level reduction in leukocytospermia? This point need to be further studied. It has important significance to reveal the mechanism of reproductive tract infection and infertility. The adjustment of PAF is affected by several factors, wherein it is related to platelet activating factor acetylhydrolase (PAF-AH). PAF-AH is specific to PAF, its concentration is negatively correlated with PAF. PAF-AH level has significant negative correlation with sperm motility in panda seminal plasma and has negative correlation with the state of sperm movement too [18]. The concentration of PAF-AH in seminal plasma of male patient, whose spinal column was injured, is prominently higher than normal people; meanwhile, it has negative correlation to sperm motility [19]. In a study investigating the relation between PAF-AH level and sperm vigor, the researchers found that when sperm motility was equal to or above 50%, PAF-AH density was 442.03 ± 14.37 IU/L, while when the vigor is below 50%, the PAF-AH density is 882.16 ± 18.45 IU/L [20]. This research also shows that PAF-AH is an important factor to adjust PAF. Whether PAF-AH plays an important role in lowering PAF level in leukocytospermia or not, it is still worthy of further study.

In conclusion, low expression of PAF or high expression of TNF-*α* in leukocytospermia affects the sperm quality, both of which contributed to sterility. The reason why PAF shows a low expression during reproductive tract infection is still not clear now, and it is of important significance, which deserves further study.

## Figures and Tables

**Table 1:** Comparison of main seminal plasma parameters between leukocytospermia group and normal group.

Seminal plasma parameter	Normal group (*n* = 15)	Leukocytospermia group (*n* = 22)
Sperm density (×10^6^/mL)	172.56 ± 28.43	170.12 ± 45.56
Sperm survival rate (%)	54.52 ± 9.67	35.86 ± 11.43
Level (a + b) sperm vigor (%)	52.31 ± 5.12	39.85 ± 19.21
Seminal plasma liquefaction time (min)	21.63 ± 4.68	29.36 ± 5.64

**Table 2:** Comparison of TNF-*α* and PAF concentration between leukocytospermia group and normal group.

Variable	Normal Group (*n* = 15)	leukocytospermia Group (*n* = 22)
TNF-*α* (ng/mL)	3.48 ± 1.08	5.51 ± 1.46
PAF (ng/mL)	6.21 ± 1.38	2.14 ± 0.43

**Table 3:** Correlation between PAF, TNF-*α*, and main seminal plasma parameters of leukocytospermia group.

		Sperm Density	Sperm Survival Rate	Sperm Vigor	Liquefaction time
TNF-*α*	Related coefficient	0.052	−0.415	−0.752	−0.430
	*P* value	>0.01	<0.01	<0.01	>0.01
PAF	Related coefficient	0.048	0.452	0.642	−0.523
	*P* value	>0.01	<0.01	<0.01	>0.01
